# Insights Into the Management of Type 2 Diabetes at Diagnosis in Spain: The NEW2TYPE2 Study

**DOI:** 10.1002/edm2.70095

**Published:** 2025-09-25

**Authors:** Concha F. García‐Prieto, Fernando Gómez‐Peralta, Rocío Villar‐Taibo, Sergio Cinza‐Sanjurjo, Jennifer Redondo‐Antón, Silvia Díaz‐Cerezo, Miriam Rubio‐de Santos

**Affiliations:** ^1^ Eli Lilly and Company, Avenida de la Industria 30, 28108, Alcobendas Madrid Spain; ^2^ Endocrinology and Nutrition Unit, Segovia General Hospital, Calle de Luis Erik Clavería Segovia Spain; ^3^ Endocrinology and Nutrition Division, Complejo Hospitalario Universitario de Santiago de Compostela, Rúa da Choupana s/n Santiago de Compostela Spain; ^4^ Epigenomics in Endocrinology and Nutrition Group, Epigenomics Unit, Instituto de Investigación Sanitaria de Santiago de Compostela (IDIS), Rúa da Choupana s/n Santiago de Compostela Spain; ^5^ Centro de Salud Milladoiro, Área Sanitaria Integrada Santiago de Compostela, Travesia Do Porto A Coruña Spain; ^6^ Instituto de Investigación Sanitaria de Santiago de Compostela (IDIS) Santiago de Compostela Spain; ^7^ Centro de Investigación Biomédica en Red‐Enfermedades Cardiovasculares (CIBERCV) Madrid Spain

**Keywords:** cross‐sectional survey, early diabetes management, glycaemic control, weight loss

## Abstract

**Introduction:**

This study analysed the opinions and perceptions of Spanish physicians towards the management of people newly diagnosed with type 2 diabetes (T2D) aged ≤ 65 years.

**Methods:**

Online survey targeting primary care physicians (PCPs) and endocrinologists (members of three national scientific societies) treating people with T2D. Management practices and factors determining prescribed therapies and treatment goals were captured in general and by patient profile. The respondents' perception of the limitations in setting strict glycaemic control objectives and weight loss targets, and the feasibility and impact of possible solutions, were examined.

**Results:**

A total of 105 physicians (60% PCPs, 40% endocrinologists) responded to the survey; 98% of respondents reported following clinical practice guidelines; 53.3% and 27.6% considered stringent glycaemic control to be HbA1c levels of 6.0%–6.5% and 6.5%–7.0%, respectively. In patient profiles with overweight/obesity, > 90% reported setting weight loss goals, with 5%–10% weight loss being the most common target. The most limiting factors for the establishment of stringent glycaemic and weight loss targets were the lack of awareness of self‐care of the disease (74.3%) and the cost to the healthcare system of the most effective drugs (72.4%). Training and the implementation of simple protocols and algorithms were the solutions perceived as having the greatest impact and feasibility. Redefining visa criteria was considered the solution with the highest impact.

**Conclusions:**

The results are consistent with clinical practice guidelines' recommendations in Spain, but early and intensive interventions focused on reducing the risk of long‐term complications in people with T2D who have longer life expectancy could be promoted at diagnosis.

## Graphical abstract

This survey of 105 Spanish PCPs and endocrinologists evaluated opinions and perceptions on the management of patients ≤ 65 newly‐diagnosed with type 2 diabetes. Most physicians follow current guidelines regarding glycaemic and weight loss goals, but some barriers remain, such as improved patient self‐care and the cost of the most effective drugs.

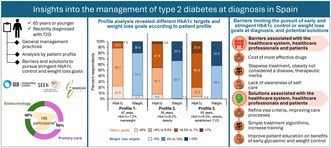

## 1 Introduction

Type 2 diabetes (T2D) is a chronic disease whose treatment requires an individualised and integrated approach to prevent long‐term complications and optimise quality of life. Currently, T2D care is based on glycaemic and weight management, and control of cardiovascular (CV) risk factors [1, 2]. Glycaemic control is usually assessed by the serum levels of glycated haemoglobin (HbA1c), which correlate to diabetes complications in the long term [3]. A reasonable HbA1c target for most people with T2D is around 7% [2], but people with longer life expectancy and those without major comorbidities may benefit from stringent HbA1c goals (HbA1c 6.5% or lower), if safely achieved, which have been proven to prevent microvascular complications later in life [4]. HbA1c goals should be pursued early after diagnosis: a better prognosis has been observed in patients who begin intensive therapy early after diagnosis than in those who achieve optimal HbA1c levels later in the course of the disease [5, 6]. Early intervention is also particularly beneficial in those with a longer life expectancy, as younger people with T2D should be considered at very high risk for complications [2]. Weight control is also central in T2D management [2, 7]. The American Diabetes Association (ADA)/European Association for the Study of Diabetes (EASD) and the European Society of Cardiology (ESC) guidelines recommend a minimum weight loss of 5%–10% to obtain metabolic improvements, while reductions of higher magnitude (10%–15%) have shown disease‐modifying effects, meaning an improved glycaemic control in the presence of a reduced need for diabetes medications or even disease remission [2, 7, 8, 9]. Weight loss has been shown to have a positive impact on HbA1c levels and is associated with a reduced risk of CV disease (CVD) [7, 10]. However, some studies have highlighted the difficulties faced by people with T2D in achieving these objectives [7].

In Spain, epidemiological investigations suggest that a significant proportion of people with T2D present with poor glycaemic control [11, 12]. Further, treatment patterns observed in clinical practice do not generally conform to the recommendations of clinical practice guidelines [13]. The recent PRIORITY‐T2D study showed that stringent HbA1c targets (HbA1c < 6.5%) were not being achieved by 46%–70% of people with T2D aged ≤ 65 years in the first 5 years after diagnosis, and that > 60% of subjects did not lose at least 5% of their baseline body weight [14]. This study also indicated that the use of the weight‐reducing drugs remained very low. Therapeutic inertia could be a cause of poor control in the years after diagnosis, especially in younger patients [15]. Although there are some studies evaluating how healthcare providers (HCPs) manage people with T2D in routine clinical practice in Spain [16, 17, 18], none focused on younger and newly diagnosed populations. Recent guidelines advocate for more intensive treatment in young patients with T2D, emphasising weight management as a key therapeutic target. However, data on their real‐world implementation remain scarce, particularly regarding how these recommendations translate into clinical practice. Understanding current management patterns, clinician adherence and potential barriers is essential to bridge the gap between guideline recommendations and actual care.

The current study investigated the attitudes and opinions of Spanish HCPs towards the care of people newly diagnosed with T2D aged ≤ 65 years. It analysed the extent to which HCPs prioritise achieving stringent glycaemic control and weight loss goals at the time of diagnosis and explored the barriers and potential solutions found by Spanish HCPs in pursuing these objectives.

## 2 Material and Methods

This was a descriptive, cross‐sectional study based on a questionnaire targeted at primary care physicians (PCP) and endocrinologists practising in Spain. Responses were collected from 18 September 2023 to 10 October 2023. Due to the nature of the study, approval by an ethics committee was not required. This study was conducted according to the ethical principles of the Declaration of Helsinki and is consistent with Good Pharmacoepidemiology Practices and applicable laws and regulations.

### 2.1 Population and Study Design

A questionnaire was designed based on a literature review and a discussion group meeting with a study Scientific Committee, integrated by two endocrinologists (F.G.P. and R.V.T.) and one PCP (S.C.S.) proposed by the medical societies endorsing the project (the Spanish Society for the Study of Diabetes [SED], the Spanish Society for Endocrinology and Nutrition [SEEN] and the Spanish Society of Primary Care Physicians [SEMERGEN], respectively) as their representatives.

To be eligible for participation, respondents were required to be PCPs or endocrinologists actively managing people with T2D within the Spanish public or private healthcare system, and members of the endorsing medical societies. Participants were recruited via the participating medical societies, which utilised their standard communication channels to distribute the survey link to all their members. Participation was voluntary and no compensation was provided for their responses. The questionnaire consisted of 41 questions and took approximately 10 min to complete, and was divided into three sections (Supporting Informations, Annex 1): Section 1 covered sociodemographic, professional and workplace characteristics of the respondents. The variables collected in this section allowed for the description of the respondent's profile and to check if inclusion criteria were met. Section 2 included items related to the management of adults aged 65 years or younger with a recently confirmed diagnosis of T2D. Open‐field, dichotomous and multiple‐choice questions were used to evaluate standard clinical practices, including self‐perceived meaning of stringent glycaemic control, clinical practice guidelines used and clinical parameters evaluated at diagnosis. This section also presented three hypothetical T2D patient profiles with different age, HbA1c control, body mass index (BMI) values and concomitant conditions to evaluate their management and determining factors: profile 1, 42‐year‐old patient, with overweight and an HbA1c of 7.2%; profile 2, 56‐year‐old patient, with obesity and an HbA1c of 8.2%; profile 3, 65‐year‐old patient, with established CVD, obesity and an HbA1c of 9.0%. To analyse the extent to which Spanish HCPs prioritise the achievement of stringent glycaemic control and body weight loss goals right after T2D diagnosis in people aged ≤ 65 years with overweight or obesity, HCPs were asked to indicate treatment goals (HbA1c and weight values targeted in each patient profile, using a free‐text field), as well as to select the most appropriate first‐line therapy for each patient profile. To investigate the main determining factors behind their decision, they were asked to distribute a total of 100 points (the higher scores indicating a greater weight of the variable in their decision, with the possibility to assigning an identical value to each option) among each of the following parameters: HbA1c, weight goals, risk or presence of complications and clinical characteristics (including age, BMI and comorbidities). Treatment options included lifestyle changes, metformin, sulfonylureas, sodium‐glucose co‐transporter 2 inhibitors (SGLT2i), pioglitazone, insulin, dipeptidyl peptidase‐4 inhibitors (DPP4i), glucagon‐like peptide‐1 receptor agonists (GLP‐1 RA) and others. Finally, Section 3 contained a list of 11 statements describing barriers and potential solutions to pursuing stringent glycaemic control and body weight loss goals in people newly diagnosed with T2D. Respondents were asked to rate each statement from 0 to 10, with 0 meaning ‘non‐limiting’ for barriers, and ‘no impact’ or ‘not feasible’ for solution impacts and feasibility.

### 2.2 Statistical Methods

To ensure the representativeness of physicians treating people with T2D in Spain, the sample size was calculated according to the number of PCPs (*n* = 36,247) and endocrinologists (*n* = 1268) who practise in the Spanish public and private healthcare system [19]. Employing a 10% margin of error and the maximum variability criterion, it was determined that a sample size of 96 HCPs would be required.

For quantitative variables, including prioritisation questions, centrality and dispersion measures (mean, standard deviation [SD]) were calculated. For qualitative variables, such as dichotomous and multi‐response questions, relative and absolute frequencies were obtained. To identify main barriers and potential solutions, relative and absolute frequencies of the perceived barriers and solutions were analysed.

## 3 Results

### 3.1 Sociodemographic and Clinical Characteristics of the Respondents

A total of 105 HCPs from all Spanish regions (except La Rioja) responded to the survey: 60% PCPs and 40% endocrinologists. The mean (SD) age of the respondents was 46 (12) years, and 64% were female. Respondents' professional characteristics are shown in Table S1. The mean (SD) years of clinical experience was 17.0 (12) and 85.7% worked in the public sector. The respondents reported attending a mean (SD) number of 24.3 (13.0) people with T2D weekly, including 2.8 (3.0) newly diagnosed cases. Of these, 42.5% were aged ≤ 65 years, and 80.1% presented with overweight or obesity.

### 3.2 General Management Practices at T2D Diagnosis

Almost all respondents reported following a clinical practice guideline (98%), with guidelines from the ADA/EASD (82%) and the Network of Groups for the Study of Diabetes in Primary Care (RedGDPS) (51%) being the most used. The SED and SEEN guidelines were also followed by a high percentage of respondents (49% and 45%).

The respondents were asked to report the percentage of patients for whom they evaluate a list of clinical parameters at T2D diagnosis (Figure S1). More than 90% of respondents reported assessing HbA1c levels, fasting glucose, lipids, serum creatinine and smoking status in > 75% of patients. Despite the majority (80%) of respondents evaluating BMI for > 75% of patients at diagnosis, waist circumference was assessed in < 24% of patients by 40% of respondents. Body composition was assessed in > 25% of patients by < 20% of respondents.

Regarding the assessment of CV risk, more than 90% of the HCPs evaluated blood pressure, smoking habits, low density lipoprotein cholesterol levels, family history of CVD, age and BMI (Figure S2). However, < 50% of survey respondents reported evaluating triglyceride levels, waist circumference and triglycerides/high density lipoprotein cholesterol ratio.

### 3.3 Glycaemic Control and Weight Loss Management at T2D Diagnosis

The mean HbA1c goal set by respondents was 6.6%. Most respondents (53.3%) considered an HbA1c between 6.0% and 6.5% as indicative of stringent glycaemic control; 27.6% considered a range between 6.5% and 7.0%; 18.1% equal to or less than 6.0%; and only 1.0% higher than 7.0% (Table 1). A large majority of respondents (82.9%) reported setting weight loss goals in newly diagnosed subjects (Table 1). The percentages of weight loss targeted varied according to the level of overweight or obesity: a greater percentage of weight loss was pursued when higher weight values were present (e.g., an objective > 10% was set by 34.3% of respondents for people with overweight and by 83.8% of respondents for people with obesity). In people with obesity class I, the majority of endocrinologists considered 5%–10% of weight loss to be sufficient, whereas PCPs considered > 10% of weight loss to be necessary. Both PCPs and endocrinologists agreed with > 10% weight loss being the recommendation for people with obesity class II (BMI 35.0 to 39.9 kg/m^2^) or III (BMI ≥ 40 kg/m^2^) (Table S2).

**TABLE 1:** Perceived meaning of stringent glycaemic control and the setting of weight loss objectives.

Variable	*N* = 105
**Definition of stringent HbA1c control, *N* (%)**	
HbA1c > 7%	1 (1.0)
6.5% < HbA1 ≤ 7%	29 (27.6)
6% < HbA1 ≤ 6.5%	56 (53.3)
HbA1c ≤ 6%	19 (18.1)
**Weight loss objectives**	
Weight loss objectives are set, *N* (%)	87 (82.9)
Weight loss objectives, %, mean (SD)	15.8 (8.3)
Overweight (25 ≤ BMI < 30 kg/m^2^), *N* (%)	
< 5%	12 (11.4)
5%–10%	57 (54.3)
> 10%	36 (34.3)
Obesity (BMI ≥ 30 kg/m^2^), *N* (%)	
< 5%	0 (0.0)
5%–10%	17 (16.2)
> 10%	88 (83.8)

Abbreviations: BMI, Body Mass Index; HbA1c, Glycated Haemoglobin; SD, Standard Deviation.

### 3.4 HbA1c And Weight Management by Patient Profile

An HbA1c objective between 6.0% and 6.5% for patient profiles 1 and 2 was set by 66.7% and 45.7% of respondents, respectively, but 60.0% set an objective between 6.5 and 7.0 for profile 3 (Table 2). The mean HbA1c targets were 6.5% for the younger profile with overweight and 7.1% for the older profile with obesity and established CVD. The age of the patient was the main factor considered when establishing an HbA1c target in both profiles 1 and 2, with scores of 55.1 and 41.5 points out of 100, respectively. For profile 3, the presence of comorbidities was the most important variable taken into consideration, with 36.0 points out of 100.

**TABLE 2:** Glycaemic and weight loss goals for each patient profile, and factors determining the decision.

Variable	Profile 1 42 years old, HbA1c = 7.2%, overweight	Profile 2 56 years old, HbA1 = 8.2%, obesity	Profile 3 65 years old, HbA1 = 9.0%, obesity, established CVD
**HbA1c targets**			
Mean % (SD)	6.5 (0.5)	6.8 (0)	7.1 (0.7)
HbA1c > 7%, *N* (%)	1 (1.0)	4 (3.8)	21 (20.0)
6.5% < HbA1c ≤ 7%, *N* (%)	11 (10.5)	44 (41.9)	63 (60.0)
6% < HbA1c ≤ 6.5%, *N* (%)	70 (66.7)	48 (45.7)	17 (16.2)
HbA1c ≤ 6%, *N* (%)	23 (21.9)	9 (8.6)	4 (3.8)
Factors determining HbA1c target^a^			
Age	55.1 (24.0)	41.5 (24.3)	24.0 (19.2)
BMI	20.4 (13.7)	28.8 (15.4)	18.0 (10.3)
HbA1c at diagnosis	24.4 (18.0)	29.7 (17.1)	22.0 (14.2)
Presence of comorbidities	0	0	36.0 (17.3)
**Weight loss goals**			
Weight loss goal set, *N* (%)	97 (92.4)	104 (99.0)	104 (99.0)
Weight loss goal, mean % (SD)	6.3 (8.2)	7.3 (12.8)	7.3 (12.8)
	6 (5.7)	1 (1.0)	0 (0.0)
5%–10%, *N* (%)	78 (74.3)	69 (65.7)	61 (58.1)
	21 (20.0)	35 (33.3)	44 (41.9)
Factors determining weight loss goals^a^			
Age	37.6 (22.6)	29.0 (15.9)	16.1 (10.2)
BMI	42.8 (20.7)	46.3 (18.2)	30.2 (14.9)
HbA1c at diagnosis	19.5 (16.9)	24.3 (15.2)	22.3 (14.8)
Presence of comorbidities	0	0	31.5 (14.8)

Abbreviations: BMI, Body Mass Index; CVD, Cardiovascular Disease; HbA1c, Glycated Haemoglobin; SD, Standard Deviation.

^a^ Respondents were asked to distribute 100 points. Mean score is provided.

More than 90% of respondents indicated that they set weight loss goals (Table 2). Generally, weight loss goals of 5%–10% were the most frequent for all patient profiles, and only 33% and 42% of respondents selected the > 10% objective in the patient profiles with obesity. When analysing factors associated with weight loss objectives, BMI was found to be the main determining factor in profiles 1 and 2, scoring 42.8 and 46.3 points out of 100, respectively, while it was the presence of comorbidities in profile 3 (31.5 points out of 100).

### 3.5 First‐Line Treatment for Each Patient Profile

**FIGURE 1:**
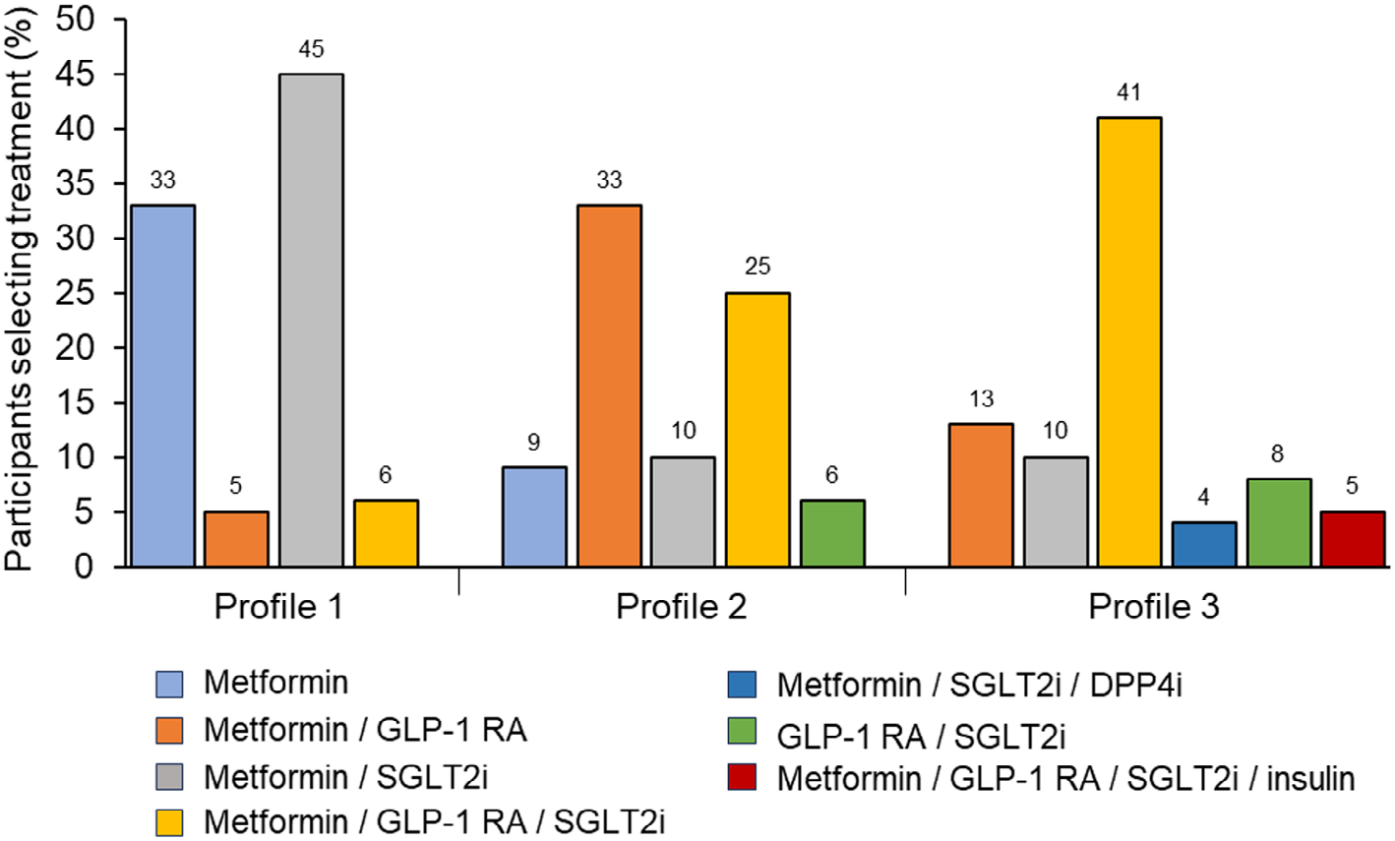
First‐line treatment for each profile. Only main options are represented in the graph. DPP4i, dipeptidyl peptidase‐4 inhibitors; GLP‐1 RA, glucagon‐like peptide‐1 receptor agonists; SGLT2i, sodium‐glucose co‐transporter 2 inhibitors.

When analysing selected options, alone or in combination, metformin was the most common first‐line treatment in all patient profiles, recommended by over 87% of respondents (Table 3). The most frequently selected first‐line treatments after metformin were SGLT2i in profile 1, GLP‐1 RA in profile 2 and SGLT2i or GLP‐1 RA in profile 3 (57.1%, 75.2% and 78.1%–79.0%, respectively). The consideration of DPP4i as first‐line treatment was low in all three patient profiles (profile 1: 3.8%, profile 2: 4.8% and profile 3: 8.6%) and the use of insulin was generally only considered for the treatment of profile 3 (10.5%). When evaluating pharmacologic treatments selected simultaneously as first‐line therapy, we observed that dual therapy was the predominant choice in profiles 1 and 2, whereas in profile 3, HCPs opted for triple therapy as the preferred first‐line treatment (Figure 1). Specifically, the most selected therapies in profile 1 were the combination of metformin/SGLT2i (45%), followed by metformin as monotherapy (33%). For profile 2, metformin/GLP‐1 RA and metformin/GLP‐1 RA/SGLT2i were the most frequently chosen options (33% and 25%). Finally, in profile 3, the use of the triple combination of metformin/GLP‐1 RA/SGLT2i was the preferred therapy (41%).

**TABLE 3:** First‐line treatment and factors determining treatment selection.

Variable	Profile 1 42 years old, HbA1c = 7.2%, overweight	Profile 2 56 years old, HbA1 = 8.2%, obesity	Profile 3 65 years old, HbA1 = 9.0%, obesity and established CVD
Lifestyle changes, *N* (%)	98 (93.3)	97 (92.4)	99 (94.3)
Only lifestyle changes, *N* (%)	1 (1.0)	0 (0.0)	0 (0.0)
Monotherapy (considering only pharmacological treatment), *N* (%)	40 (38.1)	14 (13.3)	6 (5.7)
Weight neutral diabetes medications, N (%)			
Metformin	98 (93.3)	93 (88.6)	87.6 (92)
DPP4i	4 (3.8)	5 (4.8)	9 (8.6)
Weight‐reducing diabetes medications, *N* (%)			
SGLT2i	60 (57.1)	52 (49.5)	83 (79.0)
GLP‐1 RA	14 (13.3)	79 (75.2)	82 (78.1)
Weight‐inducing diabetes medications, *N* (%)			
Pioglitazone	2 (1.9)	3 (2.9)	2 (1.9)
Sulfonylureas	0 (0.0)	0 (0.0)	3 (2.9)
Insulin	0 (0.0)	1 (0.9)	11 (10.5)
Factors determining treatment selection, %			
Glycaemic control	31.2	29.8	23.2
Weight management	23.1	32.2	23.3
Reduce the risk of complications	29.5	25.8	26.1
Clinical determinants	16.3	12.1	27.4

Abbreviations: CVD, Cardiovascular Disease; DPP4i, Dipeptidyl Peptidase‐4 Inhibitor; GLP‐1 RA, Glucagon‐Like Peptide‐1 Receptor Agonists; SGLT2i, Sodium‐Glucose Co‐Transporter 2 Inhibitors.

There were some differences in pharmacological treatments selected by the endocrinologists versus PCPs (Table S3). DPP4i was not selected by endocrinologists for any of the profiles, but was preferred by the PCPs for profile 3 (14.3% of PCPs); GLP‐1 RA was preferred by the endocrinologists for profile 2 (88.1% of endocrinologists vs. 66.7% of PCPs) and profile 3 (92.9% vs. 68.3%).

The factors determining treatment selection were glycaemic control and the reduction of the risk of complications in profile 1 (31.2 and 29.5 points out of 100, respectively) (Table 3). For profile 2, weight and HbA1c control were the most important factors (32.3 and 29.8 points, respectively). Clinical conditions and the reduction of risk of complications were prioritised (27.4 and 26.1, respectively) in profile 3.

### 3.6 Barriers and Solutions for Glycaemic Control and Weight Loss

**FIGURE 2:**
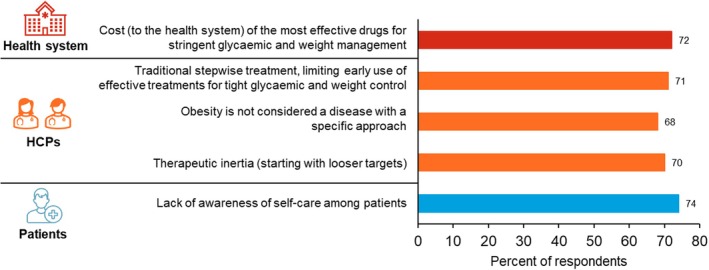
Barriers limiting the pursuit of stringent glycaemic control and weight loss goals at T2D diagnosis. Only those considered as very limiting by > 65% of respondents are shown (see Table S3 for a full list). Barriers related to the health system (red bar), physicians (orange bars) or the patients (blue bars). HCPs, healthcare providers; T2D, type 2 diabetes.

In the last section of the survey, respondents were asked to evaluate barriers and solutions to pursue stringent glycaemic control and weight loss goals at diagnosis in people aged < 65 years. More than 65% of respondents considered that the main limiting barriers (scored with 7–10) were the lack of awareness of self‐care among patients (74%), the cost to the healthcare system of the most effective drugs for the achievement of stringent HbA1c control and weight loss goals (72%), the traditional stepwise approach that limits the early use of the more effective treatments (71%), therapeutic inertia (starting with looser targets) (70%) and obesity not being considered a disease with a specific approach (68%) (Figure 2 and Table S4). The barriers associated with patients had the highest mean score (6.6, SD = 1.9), followed by barriers related to HCPs (6.2, SD = 1.8) and barriers connected to the healthcare system and guidelines (5.0, SD = 1.8) (Table S5).

**FIGURE 3:**
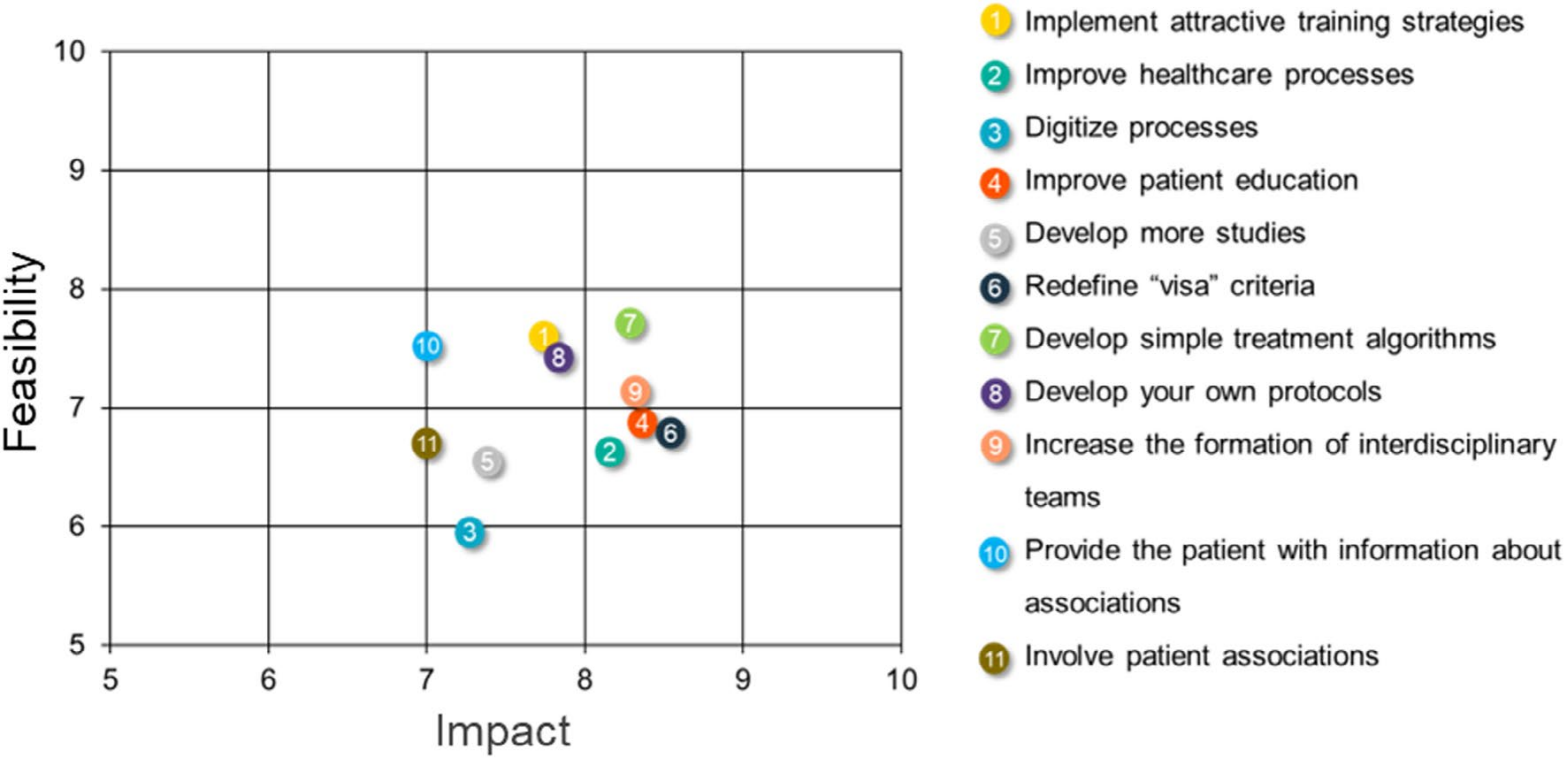
Feasibility and impact of the proposed solutions. The plot shows those solutions which scored with a mean > 5.0 in both feasibility and impact (see Table S6 for a full list of solutions).

The results of the analysis of feasibility and impact of the proposed solutions are shown in Figure 3 and Table S6. Almost all the proposed solutions were scored with a mean > 5.0 in both feasibility and impact, meaning they were considered realistic and potentially effective. The implementation of simple protocols and algorithms and the training of interdisciplinary teams for the early management of people with T2D were the solutions perceived as having the greatest impact and feasibility for overcoming barriers to pursuing stringent glycaemic and weight loss targets. Redefining visa criteria (specific medical criteria established by the health authorities in Spain that patients must meet for HCPs to prescribe certain drugs under reimbursed conditions) was a solution considered high impact by 89% of respondents, despite low feasibility. Solutions related to training and awareness of HCPs were considered of higher feasibility than impact, followed by solutions connected to the healthcare system and guidelines (Table S5). Solutions associated with patients were considered of low impact and feasibility.

## 4 Discussion

The results from this study provide valuable insights on the management practices followed by endocrinologists and PCPs when diagnosing and initiating treatment of people aged ≤ 65 years newly diagnosed with T2D.

The survey findings revealed that more than 80% of the respondents follow the ADA/EASD and RedGDPS guidelines during routine practice by pursuing an HbA1c goal between 6% and 7% [2, 20]. Consistent with weight control being considered a primary target in managing T2D, survey results showed that weight loss goals are set by most of the respondents (90%), with 5%–10% being the most common target and the minimum required weight loss to obtain metabolic improvements according to guidelines [2, 7]. However, only 33.3% and 41.9% of respondents reported pursuing > 10% weight loss objectives in patient profiles with obesity (profiles 2 and 3), despite evidence that weight losses of this magnitude have disease‐modifying effects and the population under analysis includes young people with longer life expectancy.

Regarding the selection of treatment, in general the results indicate that clinical practice guidelines are followed when selecting first‐line treatments [21]. Metformin and lifestyle changes are recommended for all three patient profiles and, as the profile's baseline clinical parameters change in terms of age, HbA1c control, BMI values and concomitant conditions, there is an increase in weight‐reducing treatments such as SGLT2i and GLP‐1 RA. Observed treatment patterns align with the recommendations in the RedGDPS, ADA/EASD, SEEN and SED guidelines, suggesting metformin as a first background therapy, and early inclusion of SGLT2i or GLP‐1 RA in patients with CV or renal risk [2, 20, 21, 22]. The choice of these treatments also matches with the main factors considered when selecting the most appropriate therapy. For example, more weight‐reducing treatments were used in profile 2, which presented with obesity, than in profile 1, which presented with overweight. The survey showed that GLP‐1 RA were preferred for a profile with obesity but without CVD (profile 2); however, in people with established CVD (profile 3), the triple combination of metformin, SGLT2i and GLP‐1 RA was the most selected therapy. This is in line with the results of a study of management of different profiles with T2D in primary care in Spain, which showed that GLP‐1 RA are preferred for people with T2D and obesity, while SGLT2i are usually chosen for patients with T2D and CVD [18].

Guidelines indicate that less rigorous HbA1c targets should be considered for people with severe comorbidities or advanced CVD [2, 8], and stricter ones (HbA1c < 6.5%) in young adult people with T2D without other CV risk factors and complications. In this respect, the survey responses showed that treatment goals according to patient profiles had stricter HbA1c targets for the younger, comorbidity‐free profile than for the older profile with established CVD. These results are consistent with the findings regarding the main factors considered when selecting first‐line treatment, as in profile 1 the main determining factor was HbA1c control, while in profile 3 it was the patient's clinical conditions. However, survey results indicate that reducing CV risk in the least complex profile is not given the priority expected when taking into account their longer life expectancy.

In Spain, the use of antidiabetic drugs with demonstrated CV benefits such as GLP‐1 RA is still low and mostly restricted to people with obesity (BMI ≥ 30 kg/m^2^) and with long‐standing and poorly controlled diabetes [11]. It is possible that weight‐lowering properties offered by GLP‐1 RA are better known among physicians than their CV and renal protection. In this study, GLP‐1 RA were significantly more often selected by endocrinologists than by PCPs for profiles 2 and 3, probably reflecting that these more complex profiles are usually treated by endocrinologists, who could tend to prescribe these highly effective drugs as first‐line therapy, in accordance with guidelines.

Waist circumference is linked to an increased risk of cardiometabolic and atherosclerotic CVD [23]. Guidelines recommend considering waist circumference when establishing weight loss goals, but the survey revealed that this parameter was measured by less than 40% of respondents in less than 24% of people with T2D at diagnosis. Further, only 43% considered this clinical parameter when estimating the CV risk, despite an 80% rate of overweight or obesity in people newly diagnosed with T2D. Considering adiposity indexes (waist circumference) and insulin resistance indicators would be beneficial for the patient according to precision medicine recommendations [24].

Despite most HCPs indicating setting weight loss goals and pursuing stringent HbA1c control, there are studies demonstrating that people with T2D in Spain are not within HbA1c control objectives, and approximately 40% of the people with T2D have poor glycaemic control [11, 12]. A recent review of observational studies concluded that there is a high clinical, economic and health‐related quality‐of‐life burden of poor metabolic and/or weight control in people with T2D in Spain [25]. Further, data from the PRIORITY‐T2D study showed that a high percentage of individuals with T2D aged ≤ 65 years were not under the more stringent glycaemic targets in the first 5 years after diagnosis, and that most people with T2D did not achieve a reduction in body weight [14]. Also, epidemiological studies have shown that obesity is highly prevalent among people with T2D in Spain, with an increasing trend [26].

Overall, the identified barriers to pursuing stringent glycaemic control and setting weight loss goals are comparable to those reported in the literature [18, 27]. Therapeutic inertia and the traditional stepwise treatment also have been highlighted as limitations in various recent studies across Spain [27, 28]. This is likely due to a lack of awareness regarding the algorithms for therapeutic intensification in T2D. Finally, as in our study, cost is often cited as a significant barrier [27]. Although the statement in this study only mentioned the cost to the healthcare system, some authors suggest that out‐of‐pocket costs are also a major barrier to accessing high‐efficacy medications and that convoluted prior authorisation processes may also contribute to the low use of adequate medication [29].

Similarly, the solutions which had the highest agreement for feasibility and impact have been proposed before [27, 30]. For example, strategies related to the patient, such as improving patient education or increasing training of interdisciplinary teams for the early management of people with T2D, have proved successful in prior studies [30]. Prior research suggests that structured education programmes have resulted in clinically significant improvements in glycaemic control, diabetes knowledge, triglyceride levels, blood pressure and medication reduction. Another solution that was highlighted in this study was to develop and implement simple treatment algorithms with clear objectives and to develop own protocols, as suggested before [27]. These studies indicate that facilitating correct decision‐making by physicians is challenging due to the amount of new information, the heterogeneity of knowledge levels and limited clinical time. Therefore, the implementation of simple treatment algorithms could improve patient care.

Survey‐based studies such as the one presented here have limitations that should be considered when interpreting the results. Selection bias cannot be excluded as this study relied on voluntary participation, and the sample may not fully represent the medical population diagnosing and treating people with T2D and aged ≤ 65 years. However, both the endocrinologists and PCPs who participated indicated substantial clinical experience in managing T2D and good knowledge and use of guidelines. Also, the survey was designed *ad hoc* for the study and lacked psychometric validation. However, it focused on objective clinical variables addressing well‐defined, routine aspects of clinical practice and, since the respondents were professionals with extensive experience in T2D management, it was estimated that the likelihood of misunderstanding or misinterpretation was low. Further, it should be highlighted that the results of the survey were based on self‐reporting and are therefore a reflection of the respondents' subjective perceptions. This could reflect medical knowledge of how they should treat specific types of patients, but not what they do in routine practice. Further, the survey captured a snapshot in time, and responses may not reflect changes occurring in clinical practice. Finally, the questionnaire presented in most cases closed, structured questions without free‐text fields where qualitative insights from respondents could be obtained.

The results of this study reveal that physicians in Spain diagnosing and treating people with T2D generally follow the recommendations of clinical practice guidelines. However, the use of adiposity indexes to qualify obesity and insulin resistance indicators was limited. Also, the management of younger and presumably less complex profiles suggests that intensive interventions focused on reducing the long‐term risk of CV complications are uncommon at T2D diagnosis. Further, the study demonstrates notable differences between the perceptions of HCPs and the degree of control of the T2D population in Spain. This information could be used to establish improved management of new‐onset T2D cases, with the overall goal of maintaining patient quality of life and avoiding disease‐related complications associated with this chronic disease. Further studies should include the perspective of patients to provide a more comprehensive view of the barriers and solutions associated with glycaemic and weight control goals.

## Author Contributions

All authors met the authorship requirements. Concha F. García‐Prieto contributed to the conception and design of the study, acquisition, analysis and interpretation of study data, drafting of the manuscript, and critical revision of the manuscript for important intellectual content. Fernando Gómez‐Peralta contributed to the design of the study, analysis and interpretation of study data, and critical revision of the manuscript for important intellectual content. Rocío Villar‐Taibo contributed to the design of the study, interpretation of study data and critical revision of the manuscript for important intellectual content. Sergio Cinza‐Sanjurjo contributed to the design of the study, interpretation of study data and critical revision of the manuscript for important intellectual content. Jennifer Redondo‐Antón contributed to the conception and design of the study, analysis and interpretation of study data, and critical revision of the manuscript for important intellectual content. Silvia Díaz‐Cerezo contributed to the conception and design of the study, interpretation of study data, and critical revision of the manuscript for important intellectual content. Miriam Rubio‐de Santos contributed to the conception and design of the study, interpretation of study data, and critical revision of the manuscript for important intellectual content.

## Conflicts of Interest

Concha F. García‐Prieto, Jennifer Redondo‐Antón, Miriam Rubio‐de Santos and Silvia Díaz‐Cerezo are shareholders and employees of Eli Lilly and Company. Fernando Gómez‐Peralta declares support for the present manuscript from Eli Lilly and Company; grants or contracts from Abbott Diabetes; and payment or honoraria for lectures, presentations, speakers bureaus, manuscript writing or educational events from Eli Lilly and Company, Abbott Diabetes, Novartis, Sanofi, Novo Nordisk, Boehringer Ingelheim Pharmaceuticals, AstraZeneca Pharmaceuticals and Bristol‐Myers Squibb. Rocío Villar‐Taibo declares payment or honoraria for lectures, presentations, speakers' bureaus, manuscript writing or educational events from Eli Lilly and Company, Novo Nordisk, Boehringer, Menarini, Dexcom, Sanofi, Abbott, Mylan and Esteve; support for attending meetings and/or travel from Eli Lilly and Company, Novo Nordisk and Menarini; and participation on data safety monitoring boards or advisory boards from Novo Nordisk. Sergio Cinza‐Sanjurjo declares receiving support for the present manuscript, consulting fees and payment or honoraria for lectures, presentations, speakers bureaus, manuscript writing or educational events from Eli Lilly and Company.

## Supporting information


**Data S1:** edm270095‐sup‐0001‐supinfo01.docx.

**Data S2:** edm270095‐sup‐0002‐supinfo02.docx.

## Data Availability

The data that supports the findings of this study are available in the [Link], [Link] of this article.
