# Correction to ‘Insights Into the Management of Type 2 Diabetes at Diagnosis in Spain: The NEW2TYPE2 Study’

**DOI:** 10.1002/edm2.70154

**Published:** 2026-01-14

C. F. García‐Prieto, F. Gómez‐Peralta, R. Villar‐Taibo, et al., “Insights Into the Management of Type 2 Diabetes at Diagnosis in Spain: The NEW2TYPE2 Study,” *Endocrinology, Diabetes & Metabolism* 8, no. 5 (2025): e70095, https://doi.org/10.1002/edm2.70095.

In Table 2, two rows under the ‘Weight loss goals’ heading, specifically < 5%, *N* (%) and > 10%, *N* (%), are missing in the published version. The revised table is provided below.

**TABLE 2:** Glycaemic and weight loss goals for each patient profile, and factors determining the decision.

Variable	Profile 1	Profile 2	Profile 3
	42 years old, HbA1c = 7.2%, overweight	56 years old, HbA1 = 8.2%, obesity	65 years old, HbA1 = 9.0%, obesity, established CVD
**HbA1c targets**			
Mean % (SD)	6.5 (0.5)	6.8 (0)	7.1 (0.7)
HbA1c > 7%, *N* (%)	1 (1.0)	4 (3.8)	21 (20.0)
6.5% < HbA1c ≤ 7%, *N* (%)	11 (10.5)	44 (41.9)	63 (60.0)
6% < HbA1c ≤ 6.5%, *N* (%)	70 (66.7)	48 (45.7)	17 (16.2)
HbA1c ≤ 6%, *N* (%)	23 (21.9)	9 (8.6)	4 (3.8)
Factors determining HbA1c target^a^			
Age	55.1 (24.0)	41.5 (24.3)	24.0 (19.2)
BMI	20.4 (13.7)	28.8 (15.4)	18.0 (10.3)
HbA1c at diagnosis	24.4 (18.0)	29.7 (17.1)	22.0 (14.2)
Presence of comorbidities	0	0	36.0 (17.3)
**Weight loss goals**			
Weight loss goal set, *N* (%)	97 (92.4)	104 (99.0)	104 (99.0)
Weight loss goal, mean % (SD)	6.3 (8.2)	7.3 (12.8)	7.3 (12.8)
< 5%, *N* (%)	6 (5.7)	1 (1.0)	0 (0.0)
5%–10%, *N* (%)	78 (74.3)	69 (65.7)	61 (58.1)
> 10%, *N* (%)	21 (20.0)	35 (33.3)	44 (41.9)
Factors determining weight loss goals^a^			
Age	37.6 (22.6)	29.0 (15.9)	16.1 (10.2)
BMI	42.8 (20.7)	46.3 (18.2)	30.2 (14.9)
HbA1c at diagnosis	19.5 (16.9)	24.3 (15.2)	22.3 (14.8)
Presence of comorbidities	0	0	31.5 (14.8)

Abbreviations: BMI, body mass index; CVD, cardiovascular disease; HbA1c, glycated haemoglobin; SD, standard deviation.

^a^ Respondents were asked to distribute 100 points. Mean score is provided.

We apologise for this error.

