# Supplementary material for: Insights Into the Management of Type 2 Diabetes at Diagnosis in Spain: The NEW2TYPE2 Study
Source: Endocrinol Diabetes Metab. 2025 Sep 25;8(5):e70095. doi: 10.1002/edm2.70095 (PMC12464348; doi:10.1002/edm2.70095)
Supplement: Supplementary file 1 — Data S1: edm270095‐sup‐0001‐supinfo01.docx. [file EDM2-8-e70095-s001.docx]

# Supplementary Materials Annex 1

**Questionnaire**

**INFORMATION SHEET**

**Rationale and context**

One of the main goals of treating type 2 diabetes mellitus (T2DM) is to reduce or normalize blood glucose levels and weight.

In this regard, the American Diabetes Association (ADA) and the European Association for the Study of Diabetes (EASD) recommend an overall HbA1c target ≤7% (or even lower if it can be safely achieved) and a minimum weight loss of 5-10% in overweight or obese patients. Strict and early control of these two clinical parameters has shown a beneficial effect on the management of complications related to T2DM and on the prognosis of the disease, which has a positive impact from a socioeconomic perspective.

Despite the recommendations and the high prevalence of the disease, there is little evidence on the extent to which healthcare professionals, in their daily clinical practice, seek strict control of these factors in adults younger than 65 years with a recent diagnosis of T2DM. Obtaining this information would optimize the care of this patient profile, improving their quality of life and reducing the risk of suffering complications related to the disease.

**Objective**

To describe the management of adults (≤65 years) with a recent diagnosis* of T2DM in Spain. The study seeks to analyse the extent to which healthcare professionals prioritise achieving strict glycemic control and weight reduction at the time the diagnosis of T2DM is confirmed, as well as to identify and describe the main barriers and potential solutions they find in their routine clinical practice to pursue these control objectives.

*Diagnosis should have occurred within the previous 2-3 months.

**Societies participating in the study**

The study has the collaboration of the Spanish Diabetes Society (SED), the Spanish Society of Endocrinology and Nutrition (SEEN), and the Spanish Society of Primary Care Physicians (SEMERGEN).

**With the endorsement of**

**With the endorsement of**

**With scientific endorsement from**


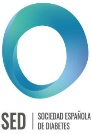

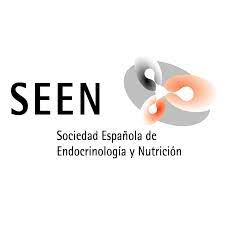

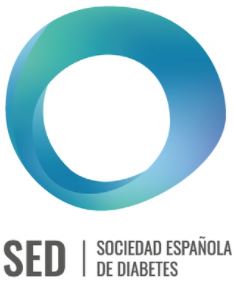


**Dr. Rocío Villar Taibo**

**Dr. Fernando Gómez-Peralta**

**Dr. Sergio Cinza Sanjurjo**


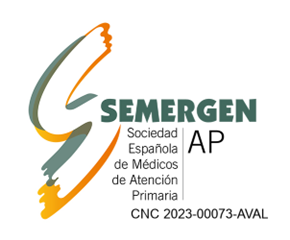


**Study design**

This cross-sectional study was based on a **survey** aimed at endocrinologists and family and community medicine specialists involved in the management of T2DM. The survey has been prepared based on a review of the literature and expert opinion, and consists of 3 sections: 1) Sociodemographic and workplace characteristics 2) Issues related to routine clinical practice; and 3) Barriers and potential strategies to promote the pursuit of strict glycemic and weight loss goals in adults with newly diagnosed T2DM.

**What does your participation consist of?**

You must answer a questionnaire, estimating a completion time of approximately **10 minutes**. The progression will be recorded, so if you are unable to complete it at the time, you can complete it whenever you wish (within the date set for completing the survey).

**Financing**

It is a project financed and carried out by Lilly, which has the methodological advice of Outcomes'10.

**Confidentiality**

The answers you provide will be anonymous since, as no personal data is collected, it will not be possible to associate them with you. In accordance with this, there will be no processing of personal data according to Regulation (EU) 2016/679 of the European Parliament and of the Council, of 27 April 2016, on the protection of natural persons with regard to the processing of personal data and on the free movement of such data, and in those aspects that it does not contemplate, Organic Law 3/2018 on the protection of personal data and guarantees of digital rights and Regulation 2016/679 of the European Parliament and of the Council will remain in force.

**I freely consent to participate in the study and consent to the access and use of my data under the conditions detailed above**

**For technical support on the platform, please contact:**

Cristina Vila – Outcomes'10

[cvila@outcomes10.com](mailto:cvila@outcomes10.com)

Telephone: 964 86 87 84

1. **Sociodemographic and clinical practice characteristics**

| *The sociodemographic variables have the sole objective of contextualizing the answers given* |
| --- |

**P01. Gender:** *[They can only check* ***one*** *option]*

□ Man

□ Woman

**P02. Age:**

___ years *[valid from 18 to 100]*

**P03. Specialty*:** *[Only one option can be checked ] [*Selection criteria P03 = "Endocrinology" or "Family and Community Medicine". If it is not fulfilled, it will be sent to the end page]*

□ Endocrinology

□ Family and Community Medicine

□ Other

**P04. Level of care of their main work center** *[They can only check* ***one*** *option]*

 First level of care (primary care centers, health centers)

 Second level of care (specialty centers and area hospitals)

 Third level of care (referral hospitals)

**P05. Autonomous Communities in which they exercise their profession** *[****fold-out*** *with the 17 Autonomous Communities and Autonomous Cities (Ceuta and Melilla, together)]*

**P06. Years of experience managing adults with T2DM (not counting years of residence):**

___ years *[valid from 1 to 50]*

**P07. Do you belong to any diabetes-related task forces?:** *[They can only check* ***one*** *option]*

□ Yes

□ No

**P08. Field in which they practice their profession:** *[They can only mark* ***one*** *option]*

□ Public

□ Private

□ Both public and private

**P09. Approximately, what number of adults with T2DM do you see weekly in your practice?**

___ [*valid from 1 to 400]*

**Q10. Approximately, what number of adults with newly diagnosed T2DM do you see weekly in your practice?**

___ [*valid from 1 to 400]*

**Q11. Of the new diagnoses of T2DM, in general, what percentage are 65 years of age or younger?**

___ % *[valid from 0 to 100]*

**Q12. Of the new diagnoses of T2DM, in general, what percentage is overweight or obese?**

___ % *[valid from 0 to 100]*

1. **Management of adults (≤65 years) with newly diagnosed T2DM**

| *Please answer the following questions based on your regular clinical practice* |
| --- |

**P13. Select the clinical practice guidelines or recommendations you use:** *[Multi-response]*

□ American Diabetes Association (ADA)/European Association for the Study of Diabetes (EASD)

□ European Society of Cardiology (ESC)/European Association for the Study of Diabetes (EASD)

□ Spanish Diabetes Society (SED)

□ Spanish Society of Endocrinology and Nutrition (SEEN)

□ Spanish Society of Family and Community Medicine (semFYC)

□ Spanish Society of Primary Care Physicians (SEMERGEN)

□ redGDPS

□ Others

□ I do not follow any clinical practice guidelines

**P14. In general, in what percentage of patients do you evaluate the following variables at the time of diagnosis?**

| **Clinical parameter** | **Percentage %** | | | |
| --- | --- | --- | --- | --- |
| Weight and height (BMI) | 0-24 | 25-49 | 50-74 | >75 |
| Waist circumference (cm) | 0-24 | 25-49 | 50-74 | >75 |
| Blood pressure | 0-24 | 25-49 | 50-74 | >75 |
| Glycated hemoglobin (HbA1c) | 0-24 | 25-49 | 50-74 | >75 |
| Fasting plasma glucose | 0-24 | 25-49 | 50-74 | >75 |
| Glucose tolerance test | 0-24 | 25-49 | 50-74 | >75 |
| Body composition analysis: dual energy densitometry, bioimpedance, etc. | 0-24 | 25-49 | 50-74 | >75 |
| Lipid Profile | 0-24 | 25-49 | 50-74 | >75 |
| Apolipoproteins | 0-24 | 25-49 | 50-74 | >75 |
| Vitamin D (ng/ml) | 0-24 | 25-49 | 50-74 | >75 |
| Serum creatinine (mg/dL) | 0-24 | 25-49 | 50-74 | >75 |
| Albumin/creatinine ratio | 0-24 | 25-49 | 50-74 | >75 |
| History of cardiovascular disease | 0-24 | 25-49 | 50-74 | >75 |
| Smoking | 0-24 | 25-49 | 50-74 | >75 |
| Alcoholism | 0-24 | 25-49 | 50-74 | >75 |
| Family history of diabetes, gestational diabetes, or insulin resistance | 0-24 | 25-49 | 50-74 | >75 |
| Microvascular complications (fundus, neuropathy examination) | 0-24 | 25-49 | 50-74 | >75 |

**P15. In general, what do you consider strict glycemic control?**

HbA1c < ____% *[valid from 1 to 10]*

**Q16 Do you generally set weight loss goals for patients newly diagnosed with T2DM?**

□ Yes

□ No

**P17 C [YES P16= IF] Please indicate the weight loss goals you would set for an adult with a recent diagnosis of T2DM who is overweight or** obese (Note: If you do not set weight loss goals for any specific profile, please indicate "0")**:**

Overweight (25 ≤ BMI <30): ____% [valid from 0 to 50]

Type I obesity (30 ≤ BMI <35): ____% [valid from 0 to 50]

Type II obesity (35 ≤ BMI <40): ____% [valid from 0 to 50]

Type III obesity (BMI ≥40): ____% [valid from 0 to 50]

**P18. Please indicate which parameters you take into account when assessing cardiovascular risk in an adult with a recent diagnosis of T2DM** *[Multiresponse]*

 Age

 Sex

 BMI

 Waist Circumference

 Glycated hemoglobin

 History of cardiovascular disease

 Family history

 LDL Cholesterol

 HDL Cholesterol

 Triglycerides

 TG/HDL

 Blood pressure

 Smoking

| *Below, we will present several profiles of adults* ***recently diagnosed*** *with T2DM in whom the health professional is considering addressing the disease for the first time. For each of the profiles, we will ask you to answer several questions based on your usual clinical practice.* |
| --- |

| **Profile 1** | A 42-year-old patient with a recent diagnosis of T2DM, HbA1c = 7.2% and overweight. |
| --- | --- |
| **Profile 2** | A 56-year-old patient with a recent diagnosis of T2DM, HbA1c = 8.2% and obesity. |
| **Profile 3** | A 65-year-old patient with a recent diagnosis of T2DM, HbA1c = 9%, obesity, and established cardiovascular disease |

HbA1c, glycated hemoglobin;

**PROFILE 1: A 42-year-old patient with a recent diagnosis of T2DM, HbA1c = 7.2% and overweight**

**P19. What glycemic control goal would you set in this case?:** *[Numeric 1-10]*

HbA1c < _____%

**P20. What aspects have had the most weight for you when setting the glycemic goal? We ask you to distribute 100 points among the given options to reflect the importance of each. The higher the score, the more important that aspect would be for you.** *[Distribution 100 points]*

Age

BMI

HbA1c at baseline

**P21. Would you set weight loss goals in this profile?** *[If the answer is YES: P22, if the answer is NO: P24]*

**□ Yes**

**□ No**

**P22 C [IF P21= IF] What weight loss goal would you set?** *[Numeric: 1-50]*

Reduction of at least _______%

**P23. What aspects have had the most weight for you when setting the weight reduction goal? We ask you to distribute 100 points among the given options to reflect the importance of each. The higher the score, the more important that aspect would be for you.** *[Distribution 100 points]*

Age

BMI

HbA1c at baseline

**P24. Which treatment would you select in the first line for this patient profile?** Multi-response, please select all the options you consider. *[Multi-response]*

 Lifestyle modifications and therapeutic education.

 Metformin

 Sulfonylureas (SU)

 Sodium-glucose cotransporter type 2 (iSGLT2) inhibitors

 Pioglitazone

 Insulin

 Dipeptidyl peptidase-4 (iDPP4) inhibitors

 Glucagon-like peptide-1 (GLP1) receptor agonists

 Other

**P25. What aspects have had the most weight for you in the selection of the chosen treatment? We ask you to distribute 100 points among the given options to reflect the importance of each. The higher the score, the more important that aspect would be for you.** *[Distribution 100 points]*

 Glycemic control

 Weight control

 Reduce the risk of developing complications or associated comorbidities

 Clinical conditioning factors of the patient (age)

**PROFILE 2: A 56-year-old patient with a recent diagnosis of T2DM, HbA1c = 8.2% and obesity**

**P26. What glycemic control goal would you set in this case?:** *[Numeric 1-10]*

HbA1c < _____%

**P27. What aspects have had the most weight for you when setting the glycemic goal? We ask you to distribute 100 points among the given options to reflect the importance of each. The higher the score, the more important that aspect would be for you.** *[Distribution 100 points]*

Age

BMI

HbA1c at baseline

**P28. Would you set weight loss goals in this profile?** *[If the answer is YES: P29, if the answer is NO: P31]*

**□ Yes**

**□ No**

**P29. C [IF P28= SI] What weight loss goal would you set?:** *[Numeric 1-50]*

Reduction of at least _______%

**P30. What aspects have had the most weight for you when setting the weight reduction goal? We ask you to distribute 100 points among the given options to reflect the importance of each. The higher the score, the more important that aspect would be for you.** *[Distribution 100 points]*

Age

BMI

HbA1c at baseline

**P31. Which treatment would you select in the first line for this patient profile?** Multi-response, please select all the options you consider. *[Multi-response]*

 Lifestyle modifications and therapeutic education

 Metformin

 Sulfonylureas (SU)

 Sodium-glucose cotransporter type 2 (iSGLT2) inhibitors

 Pioglitazone

 Insulin

 Dipeptidyl peptidase-4 (iDPP4) inhibitors

 Glucagon-like peptide-1 (GLP1) receptor agonists

 Other

**Q32 What aspects have had the most weight for you in the selection of the chosen treatment? We ask you to distribute 100 points among the given options to reflect the importance of each. The higher the score, the more important that aspect would be for you.** *[Distribution 100 points]*

 Glycemic control

 Weight management

Reduce the risk of developing complications or associated comorbidities

Clinical conditioning factors (age)

**PROFILE 3: A 65-year-old patient with a recent diagnosis of T2DM, HbA1c = 9%, obesity and established cardiovascular disease**

**P33. What glycemic control goal would you set in this case?:** *[Numerical 1-10]*

HbA1c _____%

**P34. What aspects have had the most weight for you when setting the glycemic goal? We ask you to distribute 100 points among the given options to reflect the importance of each. The higher the score, the more important that aspect would be for you.** *[Distribution 100 points]*

Age

BMI

HbA1c at baseline

Presence of comorbidities and/or established cardiovascular disease

**P35. Would you set weight loss goals in this profile?** *[If the answer is YES: P36, if the answer is NO: P38]*

**□ Yes**

**□ No**

**P36. C [IF P35= IF] What weight loss goal would you set?:** *[Numeric: 1-50]*

Reduction of at least _______%

**P37. What aspects have had the most weight for you when setting the weight reduction goal? We ask you to distribute 100 points among the given options to reflect the importance of each. The higher the score, the more important that aspect would be for you.** *[Distribution 100 points]*

Age

BMI

HbA1c at baseline

Comorbidities or established cardiovascular diseases

**P38. Which treatment would you select in the first line for this patient profile?** Multi-response, please select all the options you consider. *[Multi-response]*

 Lifestyle modifications and therapeutic education

 Metformin

 Sulfonylureas (SU)

 Sodium-glucose cotransporter type 2 (iSGLT2) inhibitors

 Pioglitazone

 Insulin

 Dipeptidyl peptidase-4 (iDPP4) inhibitors

 Glucagon-like peptide-1 (GLP1) receptor agonists

 Other

**P39. What aspects have had the most weight for you in the selection of the chosen treatment? We ask you to distribute 100 points among the given options to reflect the importance of each. The higher the score, the more important that aspect would be for you.** *[Distribution 100 points]*

 Glycemic control

 Weight management

 Reduce the risk of developing complications or associated comorbidities

 Clinical conditions (age, comorbidities, established cardiovascular disease)

1. **Barriers and Solutions to Setting a Strict Glycemic and Weight Loss Goal in Adults Newly Diagnosed with T2DM and Age ≤65 Years**

| *Next, indicate to what extent you think the following issues prevent healthcare professionals from setting strict glycemic and weight-loss targets in adults (≤ age 65) newly diagnosed with T2D.*  *(Being 0=does not prevent setting strict goals at all and 10=Completely prevents setting strict goals)* |
| --- |

Degree to which it limits healthcare professionals from setting strict glycemic and weight-loss goals

| **Barriers** | **Impact** | | | | | | | | | | |
| --- | --- | --- | --- | --- | --- | --- | --- | --- | --- | --- | --- |
| - Clinical practice guidelines with little focus on achieving strict glycemic and weight loss goals | 0 | 1 | 2 | 3 | 4 | 5 | 6 | 7 | 8 | 9 | 10 |
| - Heterogeneity in the recommendations of the different clinical practice guidelines | 0 | 1 | 2 | 3 | 4 | 5 | 6 | 7 | 8 | 9 | 10 |
| - Lack of training/awareness of the benefits of strict glycemic control in healthcare professionals | 0 | 1 | 2 | 3 | 4 | 5 | 6 | 7 | 8 | 9 | 10 |
| - Lack of awareness of the benefits of strict glycaemic control in people newly diagnosed with T2DM | 0 | 1 | 2 | 3 | 4 | 5 | 6 | 7 | 8 | 9 | 10 |
| - Lack of training/awareness about the benefit of weight management in healthcare professionals | 0 | 1 | 2 | 3 | 4 | 5 | 6 | 7 | 8 | 9 | 10 |
| - Lack of awareness of the benefit of weight management in people newly diagnosed with T2DM | 0 | 1 | 2 | 3 | 4 | 5 | 6 | 7 | 8 | 9 | 10 |
| - Therapeutic inertia (starting with more lax targets) | 0 | 1 | 2 | 3 | 4 | 5 | 6 | 7 | 8 | 9 | 10 |
| - Traditional step therapy, which limits the early use of effective treatments for strict glycemic and weight control | 0 | 1 | 2 | 3 | 4 | 5 | 6 | 7 | 8 | 9 | 10 |
| - Obesity is not considered a disease that has a specific approach | 0 | 1 | 2 | 3 | 4 | 5 | 6 | 7 | 8 | 9 | 10 |
| - Refusal of certain medical treatments by some patients. | 0 | 1 | 2 | 3 | 4 | 5 | 6 | 7 | 8 | 9 | 10 |
| - Lack of awareness of self-care of the disease by patients | 0 | 1 | 2 | 3 | 4 | 5 | 6 | 7 | 8 | 9 | 10 |
| - Cost (to the healthcare system) of the most effective drugs for strict glycaemic and weight control | 0 | 1 | 2 | 3 | 4 | 5 | 6 | 7 | 8 | 9 | 10 |
| - Difficulty in achieving strict goals with available treatments | 0 | 1 | 2 | 3 | 4 | 5 | 6 | 7 | 8 | 9 | 10 |
| - Fear of side effects of treatments (hypoglycemia, etc.) | 0 | 1 | 2 | 3 | 4 | 5 | 6 | 7 | 8 | 9 | 10 |

| *Next, you will be introduced to a series of* ***potential strategies*** *to encourage healthcare professionals to systematically establish strict goals for glycemic control and weight reduction in adults (≤ 65 years) with a recent diagnosis of DM2. For each of the strategies, you will be asked to rate the following aspects:*   - ***Impact*** *of* ***strategy implementation*** *(0= no impact and 10= major impact on establishing strict glycemic and weight control)* - ***Feasibility. Possibility of implementing each strategy in the short/medium term (<5 years)*** *(0= not at all feasible and 10= totally feasible)* |
| --- |

Degree of FEASIBILITY in 5 years

Degree of IMPACT on the establishment of strict glycemic control and weight loss

| **SOLUTIONS** | **Feasibility/ Impact** | | | | | | | | | | |
| --- | --- | --- | --- | --- | --- | --- | --- | --- | --- | --- | --- |
| - Improve patient education on the benefits of early and stringent glycaemic and weight control. | | | | | | | | | | | |
| Impact | 0 | 1 | 2 | 3 | 4 | 5 | 6 | 7 | 8 | 9 | 10 |
| Feasibility | 0 | 1 | 2 | 3 | 4 | 5 | 6 | 7 | 8 | 9 | 10 |
| - Provide information to patients about the existence of patient associations | | | | | | | | | | | |
| Impact | 0 | 1 | 2 | 3 | 4 | 5 | 6 | 7 | 8 | 9 | 10 |
| Feasibility | 0 | 1 | 2 | 3 | 4 | 5 | 6 | 7 | 8 | 9 | 10 |
| - Involve patient associations for support | | | | | | | | | | | |
| Impact | 0 | 1 | 2 | 3 | 4 | 5 | 6 | 7 | 8 | 9 | 10 |
| Feasibility | 0 | 1 | 2 | 3 | 4 | 5 | 6 | 7 | 8 | 9 | 10 |
| - Implement attractive and practical training strategies for health professionals to optimise their training process. | | | | | | | | | | | |
| Impact | 0 | 1 | 2 | 3 | 4 | 5 | 6 | 7 | 8 | 9 | 10 |
| Feasibility | 0 | 1 | 2 | 3 | 4 | 5 | 6 | 7 | 8 | 9 | 10 |
| - Develop and implement simple treatment algorithms with clear objectives according to patient profile | | | | | | | | | | | |

| **SOLUTIONS** | **Feasibility/ Impact** | | | | | | | | | | |
| --- | --- | --- | --- | --- | --- | --- | --- | --- | --- | --- | --- |
| - Develop own protocols that try to apply the guidelines in a practical way in different clinical settings | | | | | | | | | | | |
| Impact | 0 | 1 | 2 | 3 | 4 | 5 | 6 | 7 | 8 | 9 | 10 |
| Feasibility | 0 | 1 | 2 | 3 | 4 | 5 | 6 | 7 | 8 | 9 | 10 |
| - Increase training of interdisciplinary teams for the early management of people with T2D | | | | | | | | | | | |
| Impact | 0 | 1 | 2 | 3 | 4 | 5 | 6 | 7 | 8 | 9 | 10 |
| Feasibility | 0 | 1 | 2 | 3 | 4 | 5 | 6 | 7 | 8 | 9 | 10 |
| - Improving care processes | | | | | | | | | | | |
| Impact | 0 | 1 | 2 | 3 | 4 | 5 | 6 | 7 | 8 | 9 | 10 |
| Feasibility | 0 | 1 | 2 | 3 | 4 | 5 | 6 | 7 | 8 | 9 | 10 |
| - Digitise processes to facilitate patient monitoring and control (e.g. telemedicine, mobile alerts, etc.) | | | | | | | | | | | |
| Impact | 0 | 1 | 2 | 3 | 4 | 5 | 6 | 7 | 8 | 9 | 10 |
| Feasibility | 0 | 1 | 2 | 3 | 4 | 5 | 6 | 7 | 8 | 9 | 10 |
| - Develop further studies assessing the clinical, economic and social efficacy of setting stricter targets for glycaemic and weight control | | | | | | | | | | | |
| Impact | 0 | 1 | 2 | 3 | 4 | 5 | 6 | 7 | 8 | 9 | 10 |
| Feasibility | 0 | 1 | 2 | 3 | 4 | 5 | 6 | 7 | 8 | 9 | 10 |
| - Redefine visa criteria | | | | | | | | | | | |
