# Supplementary material for: Insights Into the Management of Type 2 Diabetes at Diagnosis in Spain: The NEW2TYPE2 Study
Source: Endocrinol Diabetes Metab. 2025 Sep 25;8(5):e70095. doi: 10.1002/edm2.70095 (PMC12464348; doi:10.1002/edm2.70095)
Supplement: Supplementary file 2 — Data S2: edm270095‐sup‐0002‐supinfo02.docx. [file EDM2-8-e70095-s002.docx]

**Supplementary Materials Annex 2**

**Table S1.** Professional characteristics of the respondents.

| Characteristic | Total sample  (N=105) |
| --- | --- |
| Experience (years), mean (SD) | 17 (12) |
| Participates in working groups related to diabetes, N (%) | 41 (39.0) |
| Field of activity, N (%) |  |
| Public | 90 (85.7) |
| Private | 3 (2.9) |
| Both | 12 (11.4) |
| People with T2D attended per week, mean (SD) | 24.3 (13) |
| Newly diagnosed people with T2D attended per week, mean (SD) | 2.8 (3) |
| Proportion of newly diagnosed people with T2D aged ≤65 years, % | 42.5 |
| Proportion of newly diagnosed people with T2D with overweight/obesity, % | 80.1 |
| Spanish region where the physician practises care, N (%) |  |
| Madrid | 20 (19.0) |
| Andalucía | 17 (16.2) |
| Castilla y León | 14 (13.3) |
| Cataluña | 9 (8.6) |
| Galicia | 9 (8.6) |
| Valencia | 7 (6.7) |
| Aragón | 5 (4.8) |
| Asturias | 5 (4.8) |
| Baleares | 4 (3.8) |
| Canarias | 4 (3.8) |
| País Vasco | 3 (2.9) |
| Castilla-La Mancha | 2 (1.9) |
| Extremadura | 2 (1.9) |
| La Rioja | 0 (0) |
| Murcia | 2 (1.9) |
| Cantabria | 1 (1.0) |
| Navarra | 1 (1.0) |
| Type of centre, N (%) |  |
| First level | 62 (59.0) |
| Second level | 15 (14.3) |
| Third level | 28 (26.7) |

SD, standard deviation; T2D, type 2 diabetes

**Table S2.** Perceived meaning of stringent glycaemic control and the setting of weight loss objectives.

|  | Endocrinologists  N=42 | Primary care physicians  N=63 | Total  N=105 | p-value |
| --- | --- | --- | --- | --- |
| Mean HbA1c, %, mean (SD) | 6.6 (0.3) | 6.5 (2.6) | 6.6 (0.5) |  |
| HbA1c by ranges | | | | |
| HbA1c ≤6%, N (%) | 3 (7.1) | 16 (25.4) | 19 (18.1) | 0.038 |
| 6%<HbA1c ≤7%, N (%) | 39 (92.9) | 46 (73.0) | 85 (81.0) |  |
| HbA1c >7%, N (%) | 0 (0.0) | 1 (1.6) | 1 (1.0) |  |
| Weight loss objectives | | | | |
| Weight loss objectives, yes, N (%) | 36 (85.7) | 51 (81.0) | 87 (82.9) |  |
| Weight, %, mean (SD) | 13.2 (6.1) | 17.7 (9.2) | 15.8 (8.3) |  |
| Overweight | 8.8 (8.6) | 12.0 (13.1) | 10.3 (11.6) | 0.320 |
| Obesity class (Type I–III) (BMI ≥30 kg/m^2^) | 14.9 (5.8) | 19.3 (10.7) | 17.7 (9.3) | 0.096 |
| Obesity class Type I (30≤ BMI <35 kg/m^2^) | 11.9 (6.6) | 16.8 (11.5) | 14.7 (10.0) | 0.050 |
| Obesity class Type II (35≤ BMI <40 kg/m^2^) | 14.4 (5.6) | 19.9 (11.9) | 17.6 (10.1) | 0.070 |
| Obesity class Type III (BMI ≥40 kg/m^2^) | 18.5 (8.0) | 22.2 (14.0) | 20.6 (12.0) | 0.715 |
| Weight loss objectives by ranges | | | | |
| Overall – Overweight and Obesity (BMI ≥25 kg/m^2^) | | | | |
| <5%, N (%) | 0 (0.0) | 0 (0.0) | 0 (0.0) | 0.078 |
| 5–10%, N (%) | 16 (38.1) | 14 (22.2) | 30 (28.6) |  |
| >10%, N (%) | 26 (61.9) | 49 (77.8) | 75 (71.4) |  |
| Overweight (25≤ BMI <30 kg/m^2^) |  |  |  |  |
| <5%, N (%) | 3 (7.1) | 9 (14.3) | 12 (11.4) | 0.046 |
| 5–10%, N (%) | 29 (69.0) | 28 (44.4) | 57 (54.3) |  |
| >10%, N (%) | 10 (23.8) | 26 (41.3) | 36 (34.3) |  |
| Obesity class Type I–II–III (BMI ≥30 kg/m^2^) | | | | |
| <5%, N (%) | 0 (0.0) | 0 (0.0) | 0 (0.0) | 0.914 |
| 5–10%, N (%) | 7 (16.7) | 10 (15.9) | 17 (16.2) |  |
| >10%, N (%) | 35 (83.3) | 53 (84.1) | 88 (83.8) |  |
| Obesity class Type I (30≤ BMI <35 kg/m^2^) |  |  |  |  |
| <5%, N (%) | 0 (0.0) | 2 (3.2) | 2 (1.9) | 0.033 |
| 5–10%, N (%) | 27 (64.3) | 25 (39.7) | 52 (49.5) |  |
| >10%, N (%) | 15 (35.7) | 26 (57.1) | 51 (48.6) |  |
| Obesity class Type II (35≤ BMI <40 kg/m^2^) |  |  |  |  |
| <5%, N (%) | 0 (0.0) | 0 (0.0) | 0 (0.0) | 0.205 |
| 5–10%, N (%) | 17 (40.5) | 18 (28.6) | 35 (33.3) |  |
| >10%, N (%) | 25 (59.5) | 45 (71.4) | 70 (66.7) |  |
| Obesity class Type III (BMI ≥40 kg/m^2^) |  |  |  |  |
| <5%, N (%) | 0 (0.0) | 2 (3.2) | 2 (1.9) | 0.194 |
| 5–10%, N (%) | 6 (14.3) | 12 (19.0) | 18 (17.1) |  |
| >10%, N (%) | 36 (85.7) | 49 (77.8) | 85 (81.0) |  |

BMI, body mass Index; HbA1c, glycated haemoglobin; SD, standard deviation; T2D, type 2 diabetes

**Table S3.** First-line treatment for each patient profile by specialty.

| Treatments | Profile 1.  42 yr, HbA1c 7,2% and overweight | | | Profile 2.  56 years old, HbA1=8.2%, obesity | | | Profile 3.  65 years old, HbA1=9.0%, obesity and established CVD | | |
| --- | --- | --- | --- | --- | --- | --- | --- | --- | --- |
|  | **Total** | **End** | **PCP** | **Total** | **End** | **PCP** | **Total** | **End** | **PCP** |
| Lifestyle changes, N (%) | 98 (93.3) | 38 (90.5) | 60 (95.2) | 97 (92.4) | 38 (90.5) | 59 (93.7) | 99 (94.3) | 39 (90.2) | 60 (95.2) |
| *Weight neutral diabetes medications* | | | | | | | | | |
| Metformin, N (%) | 98 (93.3) | 39 (92.9) | 59 (93.7) | 93 (88.6) | 38 (90.5) | 55 (87.3) | 0  (0.0) | 37 (88.1) | 55 (87.3) |
| DPP4i, N (%) | 4 (3.8) | 0 (0.0) | 4  (6.3) | 5  (4.8) | 0 (0.0) | 5  (7.9) | 6  (5.7) | **0**  **(0.0)** | **9 (14.3)*** |
| *Weight reducing diabetes medications* | | | | | | | | | |
| SGLT2i, N (%) | 60 (57.1) | 25 (59.5) | 35 (55.6) | 52 (49.5) | 16 (38.1) | 36 (57.1) | 83 (79.0) | 30 (71.4) | 53 (84.1) |
| GLP-1 RA, N (%) | 14 (13.3) | 8 (19.0) | 6  (9.5) | 79 (75.2) | **37 (88.1)** | **42 (66.7)*** | 82 (78.1) | **39 (92.9)** | **43 (68.3)*** |
| *Weight inducing diabetes medications* | | | | | | | | | |
| Pioglitazone, N (%) | 2 (1.9) | 0 (0.0) | 2  (3.2) | 3  (2.9) | 1 (2.4) | 2  (3.2) | 2  (1.9) | 1  (2.4) | 1  (1.6) |
| Sulfonylureas, N (%) | 0 (0.0) | 0 (0.0) | 0  (0.0) | 0  (0.0) | 0 (0.0) | 0  (0.0) | 3  (2.9) | 0  (0.0) | 3  (4.8) |
| Insulin, N (%) | 0 (0.0) | 0 (0.0) | 0  (0.0) | 1  (0.9) | 0 (0.0) | 1  (1.6) | 11 (10.5) | 5 (11.9) | 6  (9.5) |

* Statistically significant differences between specialties are marked in bold text.

CVD, cardiovascular disease; DPP4i, dipeptidyl peptidase-4 inhibitor; End, endocrinologist; GLP-1 RA, glucagon-like peptide-1 receptor agonist; PCP, primary care physicians; SGLT2i, sodium glucose co-transporter inhibitor

**Table S4.** Barriers limiting the pursuit of stringent glycaemic control and weight loss goals at T2D diagnosis. Percentage of respondents scoring in each range. Barriers which were ranked as very limiting by ≥50% of the respondents are highlighted in bold in the last column.

| **Barriers** | **Mean (SD)** | **Not limiting**  **(0)** | **Not very limiting**  **(1–3)** | **Limiting**  **(4–6)** | **Very limiting**  **(7–10)** |
| --- | --- | --- | --- | --- | --- |
| **Patient-related barrier** | | | | | |
| Lack of awareness of self-care among patients | 7.6 (2.0) | 0% | 7% | 19% | **74%** |
| Lack of awareness of the benefit of weight control in people newly diagnosed with T2D | 6.5 (2.8) | 2% | 17% | 19% | **62%** |
| Lack of awareness of the benefits of tight glycaemic control in people newly diagnosed with T2D | 6.1 (3.0) | 4% | 20% | 18% | **58%** |
| Refusal of some patients to certain medical treatments | 6.2 (2.5) | 0% | 19% | 30% | **50%** |
| **HCP-related barrier** | | | | | |
| Lack of training/awareness of the benefit of weight management among health professionals | 5.8 (2.9) | 2% | 25% | 26% | 48% |
| Lack of training/awareness of the benefits of tight glycaemic control among health professionals | 5.4 (3.0) | 7% | 22% | 27% | 45% |
| Therapeutic inertia (starting with looser targets) | 7.3 (2.4) | 1% | 10% | 19% | **70%** |
| Fear of side effects of treatments (hypoglycaemia, etc.) | 4.5 (2.5) | 7% | 32% | 39% | 22% |
| Obesity is not considered as a disease with a specific approach | 7.0 (2.4) | 1% | 10% | 22% | **68%** |
| Traditional stepwise treatment, limiting early use of effective treatments for tight glycaemic and weight control | 7.2 (2.3) | 2% | 8% | 19% | **71%** |
| **Health system and guidelines-related barrier** | | | | | |
| Cost (to the health system) of the most effective drugs for tight glycaemic and weight management | 7.1 (2.7) | 2% | 13% | 12% | **72%** |
| Difficulty in achieving stringent targets with available treatments | 5.0 (2.8) | 5% | 28% | 34% | 33% |
| Heterogeneity in the recommendations of different clinical practice guidelines | 4.3 (2.5) | 9% | 29% | 40% | 23% |
| Clinical practice guidelines with little focus on achieving stringent glycaemic and weight loss targets | 3.8 (2.7) | 17% | 29% | 40% | 14% |

HCPs, healthcare providers; SD, standard derivation; T2D, type 2 diabetes

**Table S5.** Categories of barriers and possible solutions.

|  | | Endocrinologists  N=42 | Primary care physicians  N=63 | TOTAL  N=105 |
| --- | --- | --- | --- | --- |
| *Barriers* | | | | |
| Barriers associated with patients, mean (SD) | | 6.1 (2.1) | 6.9 (1.8) | 6.6 (1.9) |
| Barriers related to training and awareness of HCPs, mean (SD) | | 6.1 (2.0) | 6.2 (1.6) | 6.2 (1.8) |
| Barriers connected with the healthcare system and guidelines, mean (SD) | | 4.6 (1.9) | 5.3 (1.7) | 5.0 (1.8) |
| *Solutions* | | | | |
| Solutions associated with patients, mean (SD) | Feasibility | 6.9 (1.9) | 7.9 (1.6) | 7.5 (1.8) |
|  | Impact | 6.9 (1.7) | 7.2 (1.7) | 7.1 (1.7) |
| Solutions related to training and awareness of HCPs, mean (SD) | Feasibility | 7.6 (1.3) | 8.4 (1.7) | 8.1 (1.3) |
|  | Impact | 7.1 (1.5) | 7.6 (1.5) | 7.4 (1.5) |
| Solutions connected with the health system and guidelines, mean (SD) | Feasibility | 7.8 (1.3) | 7.9 (1.3) | 7.8 (1.3) |
|  | Impact | 6.2 (1.4) | 6.6 (1.9) | 6.4 (1.7) |

HCPs, healthcare providers; SD, standard deviation

**Table S6.** Feasibility and impact of the proposed solutions. Percentage of respondents scoring in each range. Items in which >50% or respondents indicated that the solution had both high impact and feasibility are highlighted in bold in the last column.

| **Solutions** | | **Mean (SD)** | **No impact / not feasible (0)** | **Limited impact / not very feasible (1-3)** | **Moderate impact / feasibility (4-6)** | **High impact / highly feasible**  **(7-10)** |
| --- | --- | --- | --- | --- | --- | --- |
| **Patient-related solutions** | | | | | | |
| Improve patient education on the benefits of early and stringent glycaemic and weight control | Impact | 8.4 (1.8) | 0% | 2% | 15% | **83%** |
|  | Feasibility | 6.9 (2.3) | 0% | 10% | 29% | **62%** |
| Provide information to patients about the existence of patient associations | Impact | 7.0 (2.2) | 0% | 8% | 29% | **64%** |
|  | Feasibility | 7.5 (2.0) | 0% | 4% | 29% | **68%** |
| Involve patient associations for support | Impact | 7.0 (2.3) | 0% | 8% | 28% | 65% |
|  | Feasibility | 6.7 (2.2) | 0% | 6% | 47% | 48% |
| **HCP-related solutions** | | | | | | |
| Implement attractive and practical training strategies for health professionals to optimise their training process. | Impact | 7.8 (1.7) | 0% | 3% | 15% | **82%** |
|  | Feasibility | 7.5 (1.9) | 0% | 3% | 21% | **76%** |
| Develop and implement simple treatment algorithms with clear objectives according to patient profile | Impact | 8.3 (1.7) | 0% | 2% | 10% | **89%** |
|  | Feasibility | 7.7 (1.8) | 0% | 3% | 19% | **78%** |
| Develop own protocols that try to apply the guidelines in a practical way in different clinical settings | Impact | 7.8 (1.9) | 0% | 3% | 16% | **81%** |
|  | Feasibility | 7.4 (1.9) | 0% | 3% | 26% | **71%** |
| Increase training of interdisciplinary teams for the early management of people with T2D | Impact | 8.3 (1.7) | 0% | 1% | 12% | **87%** |
|  | Feasibility | 7.1 (2.4) | 0% | 10% | 28% | **63%** |
| **Health system and guideline-related solutions** | | | | | | |
| Improving care processes | Impact | 8.2 (1.4) | 0% | 0% | 13% | **87%** |
|  | Feasibility | 6.6 (2.0) | 0% | 8% | 40% | **52%** |
| Digitise processes to facilitate patient monitoring and control (e.g. telemedicine, mobile alerts, etc.) | Impact | 7.3 (2.0) | 0% | 5% | 25% | 70% |
|  | Feasibility | 5.9 (2.2) | 1% | 15% | 44% | 40% |
| Develop further studies assessing the clinical, economic and social efficacy of setting stricter targets for glycaemic and weight control | Impact | 7.4 (1.9) | 0% | 4% | 24% | **72%** |
|  | Feasibility | 6.6 (2.0) | 0% | 10% | 34% | **55%** |
| Redefine visa criteria | Impact | 8.5 (1.8) | 1% | 2% | 9% | **89%** |
|  | Feasibility | 6.7 (2.9) | 2% | 14% | 28% | **56%** |

HCPs, healthcare providers; SD, standard derivation; T2D, type 2 diabetes

**Figure S1.** Percentage of HCPs who evaluate the different parameters in 0–24%, 25–49%, 50–74% and >75% of patients at T2D diagnosis. The body composition analysis included densitometry and bioimpedance.

**
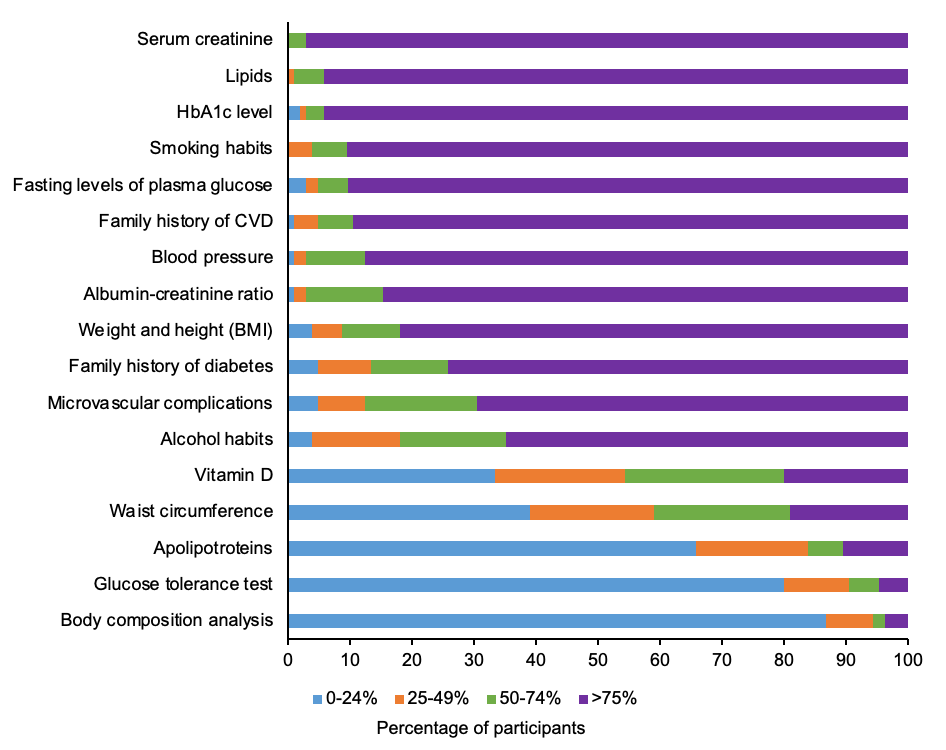
**

BMI, body mass index; CVD, cardiovascular disease; HbA1c, glycated haemoglobin; HCPs, healthcare providers; T2D, type 2 diabetes

**Figure S2.** Factors considered to assess cardiovascular risk. Percentages indicate the proportion of HCPs that evaluate these factors in people newly diagnosed with T2D. Factors that are evaluated by <50% or respondents are shown in red.

BMI, body mass index; HbA1c, glycated haemoglobin; HCPs, healthcare providers; T2D, type 2 diabetes
